# An Update on the Content of Fatty Acids, Dioxins, PCBs and Heavy Metals in Farmed, Escaped and Wild Atlantic Salmon (*Salmo salar* L.) in Norway

**DOI:** 10.3390/foods9121901

**Published:** 2020-12-19

**Authors:** Ida-Johanne Jensen, Karl-Erik Eilertsen, Carina Helen Almli Otnæs, Hanne K. Mæhre, Edel Oddny Elvevoll

**Affiliations:** 1 Norwegian College of Fishery Science, Faculty of Biosciences, Fisheries and Economics, UiT-The Arctic University of Norway, N-9037 Tromsø, Norway; karl-erik.eilertsen@uit.no (K.-E.E.); otnaes.carina@gmail.com (C.H.A.O.); hanne.maehre@gmail.com (H.K.M.); edel.elvevoll@uit.no (E.O.E.); 2 Department of Biotechnology and Food Science, Norwegian University of Science and Technology, NTNU, 7491 Trondheim, Norway

**Keywords:** Atlantic salmon, omega 3, eicosapentaenoic acid (EPA), docosahexaenoic acid (DHA), dioxins, polychlorinated biphenyls (PCB), dioxin-like PCB (dl-PCB), mercury, heavy metals, nutritional composition

## Abstract

In this paper, we present updated data on proximate composition, amino acid, and fatty acid composition, as well as concentrations of dioxins, polychlorinated biphenyls (PCBs), and selected heavy metals, in fillets from farmed (*n* = 20), escaped (*n* = 17), and wild (*n* = 23) Atlantic salmon (*Salmo salar* L.). The concentrations of dioxins (0.53 ± 0.12 pg toxic equivalents (TEQ)/g), dioxin-like PCBs (0.95 ± 0.48 pg TEQ/g), mercury (56.3 ± 12.9 µg/kg) and arsenic (2.56 ± 0.87 mg/kg) were three times higher in wild compared to farmed salmon, but all well below EU-uniform maximum levels for contaminants in food. The six ICES (International Council for the Exploration of the Sea) PCBs concentrations (5.09 ± 0.83 ng/g) in wild salmon were higher than in the farmed fish (3.34 ± 0.46 ng/g). The protein content was slightly higher in wild salmon (16%) compared to the farmed fish (15%), and the amount of essential amino acids were similar. The fat content of farmed salmon (18%) was three times that of the wild fish, and the proportion of marine long-chain omega-3 fatty acids was a substantially lower (8.9 vs. 24.1%). The omega-6 to omega-3 fatty acid ratio was higher in farmed than wild salmon (0.7 vs. 0.05). Both farmed and wild Atlantic salmon are still valuable sources of eicosapentaenoic acid and docosahexaenoic acid. One 150 g portion per week will contribute to more (2.1 g and 1.8 g) than the recommended weekly intake for adults.

## 1. Introduction

The United Nations declaration “Transforming our World: The 2030 Agenda for Sustainable Development”, with the seventeen sustainable Development Goals (SDG), emphasizes the need to achieve food safety, food security and enhanced nutrition for everybody in a sustainable manner. As land-based resources are scarce, and food production is one of the major greenhouse gas (GHG) emitters, one strategy would be shifting human diets from high carbon and GHG land-based sources (red meat) of protein to low-carbon-based sources [1,2]. The contribution of sustainable food (and feed) from well-managed ocean resources is essential.

Fish and seafood consumption has traditionally been recognized to lower the risk of cardiometabolic diseases [3,4]. This notion is primarily based on epidemiological evidence and meta-analyses [5] but also with evidence from preclinical and clinical studies on the long-chain omega-3 polyunsaturated fatty acids EPA (eicosapentaenoic acid; 20:5n-3) and DHA (docosahexaenoic acid; 22:6n-3) and lipid-soluble components present in seafood [6,7]. Even though recent clinical studies have shown variable results on cardiovascular disease risks [8], marine long-chain n-3 polyunsaturated fatty acids (PUFAs) are still regarded as cardioprotective.

Seafood is important in a balanced diet and contributes also with a wide range of other vital nutrients, such as iodine, selenium, vitamins, and high-quality proteins [9]. The nutritional recommendations to consume fish and seafood is, however mainly based on the EPA and DHA content in these foods, and the World Health Organization (WHO) recommends fish and seafood consumption to provide an average daily intake of 200–500 mg of EPA and DHA [10].

Increased attention has been drawn to the presence of persistent organic pollutants (POPs), such as dioxins and polychlorinated biphenyls (PCBs), both dioxin-like (dl) and non-dioxin-like (ndl) PCBs [11,12], along with heavy metals, such as methylmercury [13], in seafood. Dioxins and PCBs are both natural and anthropogenic toxic chemicals that accumulate in the food chain as they persist in the environment for years. Their presence in food and feed has fortunately declined in the last 30 years due to legislative measures and reducing strategies from public authorities and industry. The Stockholm Convention on Persistent Organic Pollutants, an international environmental treaty signed in 2001, including 184 parties [14], has agreed on eliminating the production of certain intentionally produced POPs and reducing or eliminating releases of unintentionally produced POPs. Further, the Codex Alimentarius Commission of the Food and Agriculture Organization (FAO) and the World Health Organization (WHO) Food Standard Programs, has established food standards, guidelines and codes of practice to prevent and reduce such contaminants in food and feed [15]. However, due to their long half-lives and persistence in nature, the abundance in the environment is still sustained, and seafood, fatty fish, in particular, is a major dietary source of these.

Atlantic salmon (*Salmo salar* L.), the main fatty fish species farmed in Europe, has traditionally been fed on feeds with high inclusion of fish oil and fishmeal from small, pelagic marine fatty fish. Because the global aquaculture production has increased and wild fish stocks are under pressure, the marine resources have become too expensive. Therefore, it has become common to replace most of the fish oils and fishmeal in the feed with terrestrial feed substitutes, and today fish oil is included at minimum levels to cover the salmon’s omega-3 PUFA requirements [16]. This is reflected in the salmon fillets, as the total level of omega-6 fatty acids has increased at the expense of omega-3 fatty acids [16]. The increased use of vegetable ingredients in fish feed has also resulted in an altered content of pollutants. Fish oil has been the primary source of POPs in Atlantic salmon, and, with the reduced content of marine oils as feed ingredients, the content of POPs has been reduced accordingly [17].

The overall health impacts of fish consumption result from the benefits of the nutrients and the counteracting risks associated with any putative pollutants present in the fish. Risk-benefit analyses of fish consumption have traditionally concluded that the benefits of seafood consumption on cardiometabolic disorders outweigh the risks [10,18]. To regulate the intake of contaminants in foods, the European Food Safety Authority (EFSA) has defined tolerable weekly intake levels (TWIs) that are regularly updated when new information becomes available. In 2018, the TWI of dioxins and dl-PCBs was lowered from 14 to 2 picograms toxic equivalents (TEQ) per kilogram of body weight per week [19] due to new knowledge regarding adverse effects on semen quality and possible effects on male fertility. As a consequence of this update, the recommended, or more precisely, tolerable, intake of fish, particularly fatty fish, needs to be and is being revised. In addition, a review of the toxic equivalency factors (TEF) of the contaminants [20] was supported by the EFSA panel of contaminants in the food chain (CONTAM panel) [19]. Salmon feed is continuously changing to meet profitability and sustainability measures and, thus, so is the fatty acid composition and content of contaminants in farmed salmon. Norway is the major producer of Atlantic salmon globally, followed by Chile, the United Kingdom, and Canada [21]. The Atlantic salmon is an international commodity, exported and consumed worldwide; thus, the nutritional composition of Norwegian salmon is of global relevance.

The present study aimed to analyze and update data on nutritional composition [22], particularly the fatty acid composition and content of dioxins, dl-PCBs, and heavy metals in farmed Atlantic salmon compared to that of wild Atlantic salmon, and, finally, to evaluate further the possible health impacts of the high inclusion of vegetable oils in the feed. As escaping from fish farms is not uncommon, farmed escapees caught in the sea were also included as a separate group in this study.

## 2. Materials and Methods

### 2.1. Fish

Farmed Atlantic salmon (*n* = 20) were obtained from Lerøy Aurora, one of the largest farmed Atlantic salmon producers in Norway, in July 2017 (farming location: Skjervøy, Norway). Wild salmon caught with pond nets at Lopphavet off the coast of Finnmark, Norway, in June 2017 and June 2018 were purchased from a local fishing company. Fifteen scale samples from each side of the fish, behind the dorsal fin and above the lateral line, were collected and sent to Norwegian Institute for Nature Research (NINA) for classification as originating from wild or farmed salmon using the methods described by Lund et al. [23] and Fiske et al. [24]. The analyses subsequently revealed that 17 of the wild fish were in fact escapees, and data on these fish were removed from the wild salmon group and analyzed as a separate study group. The farmed, wild and escaped Atlantic salmon mean gutted weight was 4.3 ± 0.3 kg, 4.3 ± 1.2 kg and 3.6 ± 0.2 kg, respectively. Within 24 h after landing or slaughter, gutted fish were manually filleted and skinned. Before homogenization in a meat mincer, the fillets were boned and trimmed for visible fat from the belly flaps and dorsal fin areas to mimic the industrial practice and for comparison with previous studies. Until analyzed, the minced fish were stored in sealed plastic bags at −50 °C. All analyses were performed on wet weight. The number of fish samples for different analyses varied depending on the cost of the analytical procedure, with fewer samples for costly methods, such as POPs and metals, compared to nutritional analyses. For these more costly methods, every other sample was selected for analyses.

### 2.2. Proximate Composition

Total lipids in the minced fillets were extracted [25] with dichloromethane/methanol (2:1, *v:v*), using heptadecanoic acid (Sigma Chemical Co., St. Louis, MO, USA) as an internal standard for fatty acids, and determined gravimetrically. Protein content was determined as the sum of individual amino acid residues (the molecular weight of each amino acid after subtraction of the molecular weight of H_2_O), using norleucine as internal standard, as described previously [26]. Water and ash contents were determined using the Association of Analytical Chemists (AOAC) 925.04 and AOAC 938.08 methods [27].

### 2.3. Fatty Acid Composition

Fatty acid composition was determined by gas chromatography (GC-FID) of fatty acid methyl esters (FAMEs) as previously described [28] after dissolving the extracted lipids (10 mg/mL) in dichloromethane/methanol (2:1, (*v:v*)) before methylation [29].

### 2.4. Amino Acid Composition

The amino acid composition was analyzed by dissolving approximately 200 mg of fish samples in 0.7 mL distilled H_2_O and 0.5 mL 20 mM norleucine (internal standard) and hydrolyzed as previously described [26,30]. Following hydrolysis, 100 µL aliquots of the hydrolysates were evaporated under nitrogen gas until complete dryness and re-dissolved to a suitable concentration in lithium citrate buffer at pH 2.2. All amino acids were analyzed chromatographically using an ion exchange column followed by ninhydrin post column derivatization on a Biochrom 30 amino acid analyzer (Biochrom Co., Cambridge, UK). Amino acid residues were identified using the A9906 physiological amino acids standard (Sigma Chemical Co., St. Louis, MO, USA) as described previously [28].

### 2.5. Dioxins and Furans, PCBs, and Metals

Samples were analyzed for polychlorinated dibenzo-p-dioxins (PCDD), including 2,3,7,8-tetrachlorodibenzo-p-dioxin (TCDD), 1,2,3,7,8-pentachlorodibenzo-p-dioxin (PeCDD), 1,2,3,4,7,8-hexachlorodibenzo-p-dioxin (HxCDD), 1,2,3,6,7,8-HxCDD, 1,2,3,7,8,9-HxCDD, 1,2,3,4,6,7,8-heptachlorodibenzo-p-dioxin (HpCDD) and octachlorodibenzo-p-dioxin (OCDD) and polychlorinated dibenzofurans (PCDF), including 2,3,7,8- tetrachlorodibenzofuran (TCDF), 1,2,3,7,8-pentachlorodibenzofuran (PeCDF), 2,3,4,7,8-PeCDF, 1,2,3,4,7,8-hexachlorodibenzofuran (HxCDF), 1,2,3,6,7,8-HxCDF, 1,2,3,7,8,9-HxCDF, 2,3,4,6,7,8-HxCDF, 1,2,3,4,6,7,8-heptachlorodibenzofuran (HpCDF), 1,2,3,4,7,8,9-HpCDF, and octachlorodibenzofuran (OCDF). The non-ortho polychlorinated biphenyls (PCB) analyzed were PCB 77, PCB 81, PCB 126, and PCB 169, and the mono-ortho PCBs analyzed were PCB 105, PCB 114, PCB 118, PCB 123, PCB 156, PCB 157, PCB 167, and PCB 189. Further, PCB 28, PCB 52, PCB 101, PCB 138, PCB 153, and PCB 180, which are commonly referred to as ICE-6 PCB, were also analyzed. These congeners have been assigned a WHO-TEF2005 and are included in the current EU maximum limit [20]. The dioxins, dl-PCB and six ICES (International Council for the Exploration of the Sea) PCB (ICES-6 PCB) were analyzed by ALS Laboratory Group Norway AS applying international protocols of analysis (US EPA 1613 [31] and US EPA 1668 [32], based on appropriate sample clean-up and determination by high-resolution gas chromatography/high-resolution mass spectrometry (HRGC-HRMS), using the isotope dilution method, as specified by the EU Regulation 1883/2006/EC. The laboratory has been accredited according to ISO/IEC 17025.. Mercury was analyzed by AkvaplanNiva by a direct mercury analyzer of total mercury (Milestone DMA-80, 660-1660 terminal with DMA-80 PC software quartz boat) by a method based on the EPA method 7473 [33]. The laboratory was accredited according to ISO/IEC 17025. Lead, arsenic and cadmium were analyzed by Fera Science Limited. The laboratory has been accredited according to ISO/IEC 17025.

### 2.6. Statistics Description

The results are presented on wet weight as arithmetic mean of 10–23 parallels ± standard deviation (SD). Statistical analyses were performed using the Statistical package for the social sciences v. 25 (SPSS Inc., Chicago, IL, USA). Shapiro-Wilk’s test for normality and Levene’s test for homogeneity of variance were performed, and one-way analysis of variance (ANOVA) was performed on normally distributed. For non-normally distributed data, the non-parametric Mann–Whitney U test was applied. For evaluation of statistics, Tukey and Dunnett’s T3 post-hoc tests were run for equal and unequal variances, respectively. Variables with *p* < 0.05 were considered significantly different.

## 3. Results

The gutted weights of wild and farmed Atlantic salmon were similar: 4.3 kg, whereas escapees were significantly smaller: 3.6 kg (Table 1). Wild Atlantic salmon tended to be longer than both farmed and escaped salmon, but, due to individual variation within the group, this was not significant. The condition factor varied between all groups, being highest in farmed salmon and lowest in wild salmon.

The proximate composition of the fillet of wild, farmed, and escaped salmon, is shown in Table 2. The fat content of farmed and escaped farmed salmon was three times and twice that of wild salmon (18, 12, and 6%, respectively). The protein content was significantly higher (although the numerical difference was small; 16 and 15%, respectively) in wild salmon compared to farmed fish (16 and 15%, respectively). The escaped salmon had intermediate protein content. The water content followed the fat content inversely being highest in wild salmon (70%) compared to farmed salmon (61%) and escaped salmon (67%).

### 3.1. Lipids

The fatty acid composition (% of total fatty acids) and the total amount of fatty acids per 100 g of fillets of wild, farmed, and escaped salmon, are presented in Table 3. Lipids in wild salmon contained 20.8% saturated fatty acids (SFA), 46.6% monounsaturated fatty acids (MUFA), and 31.0% polyunsaturated fatty acids (PUFA), whereas the values for farmed and escaped farmed salmon were 15.1, 40.8, and 41.9% and 14.2, 50.6, and 32.8%, respectively. The lipid concentration of EPA and DHA were significantly higher in wild salmon (6.7 and 14.6%) compared to farmed (2.6 and 4.9%) and escaped salmon (2.6 and 5.6%), whereas linoleic acid and alpha-linolenic acid were more abundant in farmed salmon (14.4 and 10.3%) compared to escaped (12.8 and 5.3%) and wild (1.4 and 1.0%) salmon. The ratio between omega-6 and omega-3 (n6/n3) fatty acids was significantly lowest in wild salmon (0.05) compared to farmed and escaped farmed salmon (0.7 and 0.8, respectively). The total content of EPA was slightly higher in farmed salmon (0.5 g/100 g) than wild salmon (0.4 g/100 g), mainly stemming from a higher lipid content in the farmed fish. The EPA content of escapees was lower (0.3 g/100 g). This trend was also seen for DHA, slightly higher in farmed (0.9 g/100 g) compared to wild salmon (0.8 g/100 g), whereas DHA in the escapees was lowest (0.7 g/100 g). The content of linoleic acid (LA), on the other hand, was significantly higher in farmed salmon fillet (2.5 g/100 g) compared to escapees (1.6 g/100 g) and wild salmon fillet (0.1 g/100 g).

### 3.2. Amino Acids

**Figure 1:**
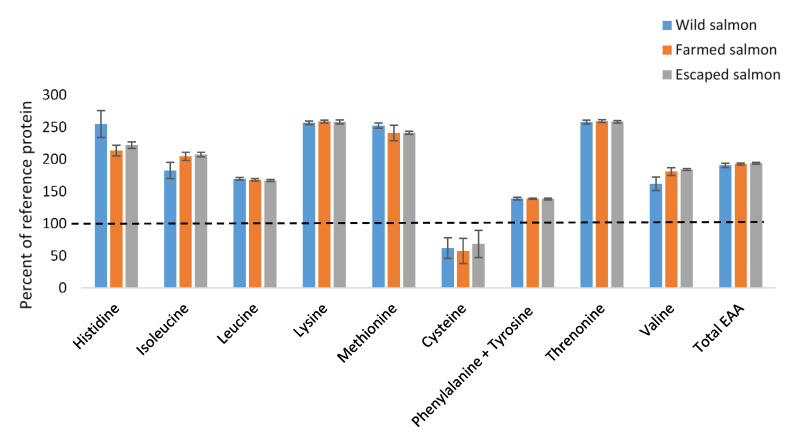
The essential amino acids (percent coverage) in protein of wild, farmed or escaped Atlantic salmon (*Salmo salar* L.) as compared to estimated requirements in adults (mg/g protein). The dotted line is included at 100% coverage [34].

The amino acid composition of wild, farmed and escaped Atlantic salmon are presented in Table 4. Glutamic acid was the most abundant amino acids for all groups, with approximately 27–29 mg/g fillet, followed by lysine (approximately 18 mg/g), leucine (approximately 16 mg/g) and aspartic acid (approximately 15 mg/g). Wild salmon had a higher content of all essential amino acids, except isoleucine and valine. The amount of essential amino acids per g protein was similar between all groups and, except for cysteine, also higher than the reference protein (Figure 1). The sum of essential amino acids was not significantly different between the groups.

### 3.3. POPs and Metals

**Figure 2:**
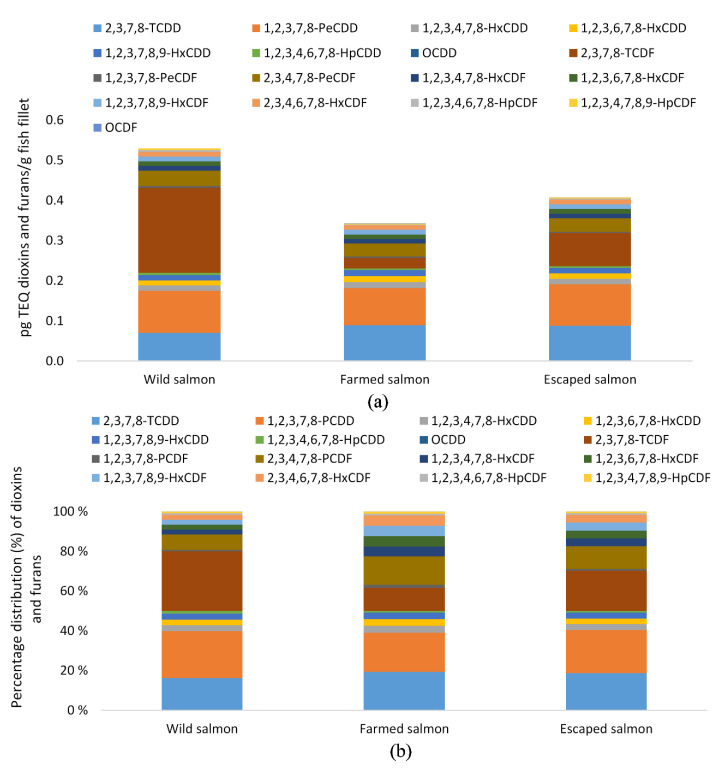
(**a**) Distribution and (**b**) percentage distribution of dioxins and furans (pg toxic equivalents (TEQ)/g fillet) in wild (*n* = 12), farmed (*n* = 10), and escaped (*n* = 10) Atlantic salmon.

**Figure 3:**
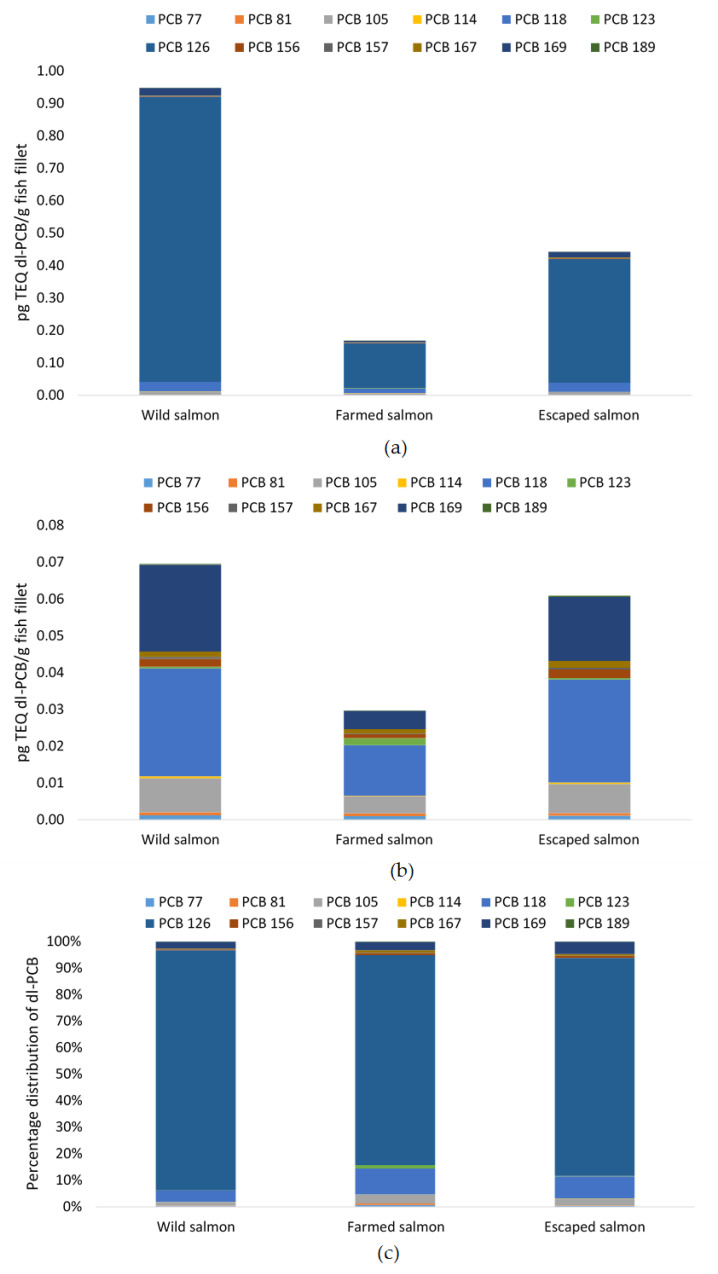
(**a**) Distribution of dl-polychlorinated biphenyls (PCB) (pg toxic equivalents (TEQ)/g fillet) including PCB 126, (**b**) distribution of dl-PCB (pg TEQ/g fillet) excluding PCB 126, and (**c**) percentage distribution of dl-PCB, in fillets of wild (*n* = 12), farmed (*n* = 10), and escaped (*n* = 10) Atlantic salmon.

**Figure 4:**
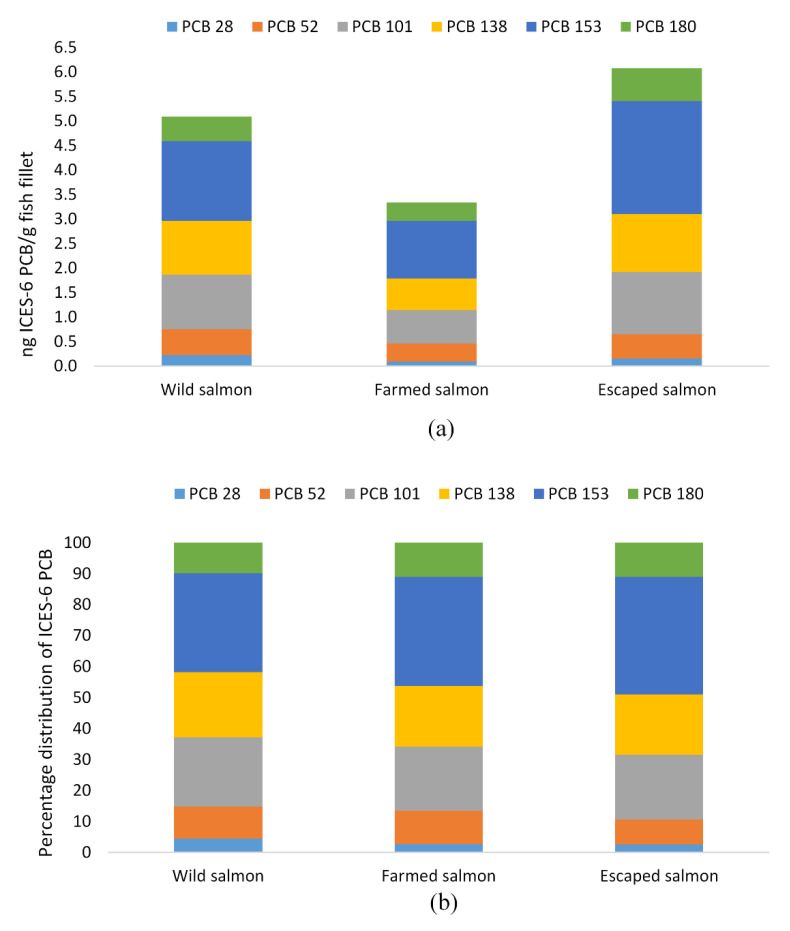
(**a**) Distribution and sum and (**b**) percentage distribution, of distribution of the six ICES (International Council for the Exploration of the Sea) PCBs (ICES-6 PCB) in fillets of wild (*n* = 12), farmed (*n* = 10) and escaped (*n* = 10) Atlantic salmon (*Salmo salar* L.).

The highest dioxins and furan levels and dl-PCB levels were found in wild salmon, with levels of 1.48 pg TEQ/g fillet compared to 0.57 and 0.9 pg TEQ/g fillet of farmed and escaped salmon, respectively (Table 5). The level of ICES-6 PCB was also significantly higher in wild salmon compared to its farmed counterpart (5.1 and 3.4 ng TEQ/g fillet, respectively) and highest in escapees (6.1 ng TEQ/g fillet). From the distribution of dioxins and furans, it was evident that the significant contribution came from a few congeners (Figure 2a,b). Whereas the percentage distribution of dioxins was similar between the three species, this was not the case for furans. The congeners 2,3,7,8 TCDD and 1,2,3,7,8 PCDD were the major dioxin constituents, accounting for 16 and 24% of the dioxins and furans in wild Atlantic salmon and 19 and 21% in both farmed and escaped salmon (Figure 2b). The 2,3,7,8 TCDF was the principal furane constituent and differed significantly between all groups. The congener’s content was 0.2 pg TEQ/g in wild, 0.03 and 0.08 pg TEQ/g in farmed and escaped Atlantic salmon, respectively, representing 30, 12, and 20% of sum dioxins and furans. The second highest content of congener of the furans, 2,3,4,7,8 PCDF, contributed with 8, 14, and 11%. Of the dl-PCBs, the non-ortho congener PCB 126 was significantly different between all three groups (0.9, 0.1 and 0.4 pg TEQ/g fillet). It accounted for 90, 80, and 82% of the total sum of dl-PCB in fillets of wild, farmed and escaped farmed salmon, respectively (Figure 3). When excluding this congener, PCB 118 and PCB 169 were major contributors among the dl-PCB. The distribution of ICES-6 PCB (Figure 4a,b) showed that PCB 153 constituted more than 30% of the sum ICES-6 PCB in all groups, whereas PCB 101 and PCB 138 accounted for approximately 20% each.

The elements mercury, arsenic, lead, and cadmium were analyzed in this study. No specification analysis of mercury was conducted, and it was, therefore, appraised as if all mercury present is methylmercury. The mercury and arsenic concentrations were significantly different between all groups. They were highest in wild Atlantic salmon (56.6 µg/kg and 2.5 mg/kg fillet, respectively), lowest in farmed salmon (18.1 µg/kg and 0.86 mg/kg fillet, respectively), and intermediate in escaped farmed salmon (34.9 µg/kg and 1.68 mg/kg fillet, respectively) (Table 4). The concentrations of lead and cadmium were lower than detection limits for all groups and set to 0.01 mg/kg.

## 4. Discussion

The study was initiated to analyze and update data on proximate composition, precisely, the amino acid composition, fatty acid composition and content of dioxins, dl-PCBs, ICES-6 PCB, and selected metals in farmed Atlantic salmon (*S.salar)* compared to wild Atlantic salmon. As a considerable proportion (43%) of the wild fish indeed was shown to be farmed escapees caught at sea, they were included as a separate group in the study. Further, we wanted to evaluate the possible health impacts of the high inclusion of vegetable oils in the feed.

Wild Atlantic salmon is both culturally and economically important in Norway. Recently, escaped farmed salmon from aquaculture was identified as the most apparent threat to the wild salmon populations [35]. According to the national surveillance program (in rivers), a wild salmon population is considered critically endangered if the proportion of farmed salmon is >10%. The proportion of escapees reported among wild spawning populations has decreased from 20–35% across monitored populations before 1998, to a 9–18% level after 2003 [35,36]. There is less data available on the proportion of escapees in the sea fisheries. Lundebye et al. [37] found 12% escapees in their scattered sampling in fisheries in 2012. Compared to their results, our results, representing two single samplings, were staggering high. The most plausible explanation is that this is a result of major escape incidents in the region. According to both fatty acid analyses and scale analyses, most of the escapees had spent less than a year in the sea [38].

As expected, differences in gutted weight, length, and condition factor were observed between the groups. The distribution of age and size of slaughtered farmed salmon from a production cycle tends to be more homogenous than that of wild (and escaped) salmon harvested at sea. This is reflected by larger variation in the latter group(s).

Observed differences in lipid content, fatty acid composition, and contaminants between wild, farmed, and escaped Atlantic salmon are caused by the different diets and feeding strategies between the three salmon groups.

### 4.1. Lipid Content

As expected, the most striking difference between farmed and wild salmon was the fat content three times higher in farmed salmon than its wild counterpart. This reflects intensive production and typical commercial conditions, and the abundant supply of energy dense feed and salmon confinement in net pens adds to fat accumulation in salmon flesh because of the reduced activity levels. The escapees had a fat content between farmed and wild salmon, which are caused by the sudden need for the escapees to adapt to a wild diet (in addition to higher activity levels in the wild) [39].

Compared to our previously reported results on the lipid content of flesh from Atlantic salmon, it is evident that the lipid content of farmed salmon has increased from 12.3% (2010) to 17.9% (2017/2018). In contrast, the fat content in wild Atlantic salmon fillets is relatively stable (6.3% in 2010 [22] and 6.0% in 2017/2018) (at least during the capture season). The fat content of farmed salmon was twice in 2010 and three times in 2017, compared to wild salmon (12%, 18%, and 6%, respectively). Similar results have also been published previously for other Atlantic salmon populations [16,37,40,41,42,43,44]. For the consumers, the increased fat content of farmed Atlantic salmon contributes to increased energy intake. Results from the population-based Norwegian studies conducted using data from two cross-sectional surveys (Tromsø 4 and 6, http://tromsoundersokelsen.no), with data from 4528 individuals and 13 years follow-up, showed that individuals consuming fatty fish at least once per week had increased waist circumference compared with those eating fatty fish less than once a week [45]. When reaching almost 18% fat content, higher than most industrial muscle foods, it might be a concern when consumers select fish for specific health benefits, especially for individuals suffering from overweight and obesity.

### 4.2. Fatty Acid Content

The high content of LA (18:2n-6) and alpha-linolenic acid (ALA) (18:3n-3) in the farmed salmon illustrates the substantial inclusion of vegetable oils in the feed. The high amount of LA in some wild-caught salmon indicated that these individuals were escaped farmed salmon. The scale analysis confirmed this notion. The content of LA in the farmed salmon was almost four times higher compared to our results from 2010 [22] and 20 times higher compared to wild salmon. The dramatic change for this particular fatty acid is explained both by the general increase in muscle lipid content and the relative increase of LA in the lipids. The content of ALA was nine times higher in the present study compared to our results from 2010 [22]. Due to changes in price and availability of marine resources, the feed composition has changed markedly from 1990 until today, from mainly marine ingredients to plant ingredients mainly [40]. The content of carbohydrate sources or binders and micronutrients, vitamin and mineral mixes, phosphorus, astaxanthin, and amino acids has remained stable (carbohydrates accounting for 10.6% and micronutrients accounting for 4% of the salmon feed in 2016 [43]). Marine protein sources constituted 14.5% of the feed in 2016, (decreased from 65.4% in 1990 and 25% in 2010) and marine oils constituted 10.4% of the feed in 2016 (24% in 1990, 16.6% in 2010). A corresponding growth in the inclusion of vegetable oils from no vegetable oils in 1990, via 12.5% in 2010 to 20.2% in 2016, was observed [37,40,43]. Although plant oils are suitable alternatives to fish oil in fish feed, the reduced inclusion of marine oils is evident by the reduced percentages of both EPA and DHA in the fillet fat. These fatty acids are now provided in the fish feed mainly to cover the salmon’s minimum requirements [46]. The content of long-chain omega-3 PUFAs in the fillets of farmed salmon was one third compared to wild salmon. In the farmed salmon, EPA and DHA were reduced from 5.5 and 8.4% to 2.6 and 4.9%, respectively, compared to our previous report [22]. Atlantic salmon is the dominant salmon species in aquaculture globally, and a small number of international feed companies produce feed for farmed salmon in Norway, Chile, Scotland, Ireland, and the Faroe Islands. Feed formulations likely differ slightly from one country to another (and from one company to another), yet the trends in feed development are similar, at least regarding the macronutrient compositions.

### 4.3. Nutritional Evaluation of Omega-3 Fatty Acids and Essential Amino Acids in Salmon

The nutritional quality of proteins depends on the presence and concentration of essential amino acids [47]. Nine amino acids are essential to humans, i.e., they are not synthesized in sufficient amounts and need to be obtained through the diet. All essential amino acids were present in high amounts in the protein of both wild and farmed salmon and the protein is thus of high quality [34]. The differences in the amino acid profiles reflect only minor variations in muscle proteins, indicating that intensive feeding may slightly influence the protein structure and composition of farmed salmon. Health authorities generally recommend that people consume oily fish regularly to promote long-chain PUFAs [6]. Our nutritional quality evaluation of fish omega-3 PUFA compositions, stresses the importance of taking the total lipid content into account. With this in mind, a recommended or adequate intake of 0.250 g EPA and DHA per day [48] can still be covered by consuming a small portion (20 g) of both wild and farmed salmon, whereas a 150 g portion meets the recommendations for EPA and DHA for a week (seven, eight, and six days for wild, farmed, and escaped farmed salmon, respectively). Consumption of wild salmon has advantages due to its lower fat and energy content and higher EPA and DHA concentrations. However, as wild salmon is more expensive, season dependent, and thus not easily available for all consumers, farmed salmon has become a more relevant alternative, being used in various meals (breakfast, lunch, and dinner, served raw, baked, or smoked).

In addition to increasing the dietary intake of long-chain omega-3 PUFAs, lowering the intake of omega-6 PUFAs has been considered beneficial to human health. The omega-6 to omega-3 ratio of the present Western diet has been calculated to be as high as 15–17/1 [49]. Even though our results showed that the ratio of omega-6 to omega-3 of farmed Atlantic salmon was more than ten times higher (0.7 vs. 0.05) than that of wild salmon, and has increased from 0.4 in 2010, it is still below 1. Thus, farmed Atlantic salmon will contribute positively by lowering the omega-6 to omega-3 ratio of the diet.

### 4.4. Contaminants

Dioxins (PCDD/Fs) and dl-PCBs are known to cause adverse effects on the immune, endocrine and nervous systems, and impairing reproductive function and may cause cancer [50]. In 2018, the European Food Safety Authority (EFSA) performed a revision of human epidemiological studies and experimental animal trials. Based on a critical effect on semen quality, EFSA updated the TWI for dioxins and dl-PCBs, to 2 pg TEQ/kg/week, which is seven times lower than the previous TWI (14 pg TEQ/kg/week) [19]. Both the Norwegians diet and the content of contaminants in Norwegian fish are closely surveyed, and, as for most Europeans, fish and other seafood is a significant contributor to the exposure of dioxin and dl-PCB [19]. Fish and seafood, followed by meat and meat products (9–34%) and milk and dairy products (7–25%), was the food category with the most substantial contribution (30–75%) to the total upper bound (UB) exposure in most population groups [50]. The exposure to dioxins and dl-PCBs in adults in Norway [18,51] was calculated to mean, lower bound (LB), exposure from all fish species to 1.4 pg TEQ/kg bw/week, while the mean, UB exposure was 1.7 pg TEQ/kg bw/week. As expected, fatty fish was the main source, contributing 76% of all fish dioxins and dl-PCBs and farmed salmon contributed 36% of this. The shift of farmed salmon feed ingredients from marine to vegetable ingredients has steadily reduced the content of dioxin and dl-PCB, and thus the contribution from salmon is presumably steadily decreased. Even if the content of dioxins and dl-PCBs in the wild salmon is three times higher than in farmed fish, wild salmon’s contribution is negligible or less than 1% [18]. Wild salmon’s low contribution may be explained by seasonality, price, and relatively low availability, even in Norway [52]. As escapees are unintended and have grown with the growth in farming, to generally 5–10% and sold as wild fish, the contribution is negligible. Obviously, the consumption of other food than fish may lead to additional dietary exposure to dioxins and dl-PCB.

This study showed that wild salmon had the highest average concentrations of dioxins, furans, and dl-PCB, whereas escaped farmed salmon had the highest content of ICES-6 PCB. The uptake of dioxins and PCBs by fish occurs both via gills and diet. Both the initial diet and farming location of the escapees are unknown. The three groups’ diets could be very different, and different prey/diet components may differ substantially for any group of compounds. All species had a content of dioxins and dl-PCB and ICES-6 PCB well below the EU maximum levels of 6.5 and 75 µg TEQ/kg fish filet, respectively. The capital contributors to the sum of dioxins and dl-PCB were the non-ortho congeners and PCB 126 that constituted 60% of dioxins and dl-PCBs in wild salmon and 45 and 27% of dioxins and dl-PCBs in farmed and escaped salmon, respectively. As for salmon in this study, PCB-126 is the dl-PCB contributing most to the current intake of PCDD/Fs and DL-PCBs. Concerning the critical effect of dioxins on semen quality, no association was observed when including dl-PCB-TEQ. This is also supported by in vitro experiments with human cells indicating that PCB-126 is less potent in humans than a TEF of 0.1 suggested by the WHO 2005 [53]. Thus, to improve the relevance of such assessment for humans, EFSA recommended that WHO2005-TEFs should be re-evaluated, and more knowledge about the relative potency of PCB-126 is needed. In particular, this may interfere when the concentration of PCB126 is high.

Our results confirm that mercury and arsenic levels are lower in farmed than in wild salmon, as previously presented by Lundeby et al. [37]. The mercury and arsenic concentrations were, like dioxins and PCBs, highest in wild salmon, lowest in farmed salmon and intermediate in escaped salmon. The lower concentration in farmed Atlantic salmon is likely due to the high inclusion of vegetable ingredients, whereas the intermediate concentrations in escapees is a logical consequence of a shift to a marine diet. The concentrations in farmed Atlantic salmon were lower than data reported from 2015 [37] and data from the period 1999–2011 [17] but higher than data reported from Canada [44], where the inclusion of vegetable ingredients was higher (the n-6/n-3 ratio was twice as high) compared to this study. Seafood is considered the main source of dietary arsenic. However, as the main arsenic species is arsenobetaine, it is not considered toxic. Mercury, on the other side, is one of the most toxic elements, and it is estimated that 80–100% of mercury in fish is present as methylmercury [54]. The mercury concentrations were nine-fold lower than the EU maximum level of 0.5 mg MeHg/kg fillet [55]. The concentrations of lead and cadmium in all groups were lower than the limits of quantification and thus set to a value of 0.01 mg/kg filet. This value is well below EU maximum levels for these metals in fish (0.05 mg Cd/kg and 0.3 mg Pb/kg fillet of most fish species [55]. Lundeby et al. [37] reported similar results for lead but lower cadmium levels.

### 4.5. Future Prospects

As mentioned, the UN 2030 Agenda emphasizes the need for food security and improved nutrition.

It is anticipated that sustainable well-managed ocean resources will contribute through dietary shifts from land-based protein sources towards marine protein sources [1]. While capture fisheries have stagnated, aquaculture has demonstrated its food security role, growing 7.5% per year since 1970 [56]. One of the conclusions in the United Nations Food and Agriculture Organization (FAO) report, The State of World Fisheries and Aquaculture, was that aquaculture has increased fish availability in regions with limited access to fish, leading to improved nutrition and food security [56]. Although salmon, in general, is not perceived as a large contributor to global food security, the technological and biological innovations associated with salmon farming (feeds, genetic selection, biosecurity, and disease control) are used in improving fish farming in general and thereby also food security and nutrition, in particular. The growth in the production of farmed salmon and the environmental challenges imposed by such intensified production have demanded new developments in, among other fields, feeds with associated effects on both content of contaminants and nutrients. This has spurred a search for sustainable, abundant, unexploited, preferentially lower trophic levels, nutrient-dense biomass from the ocean for feed. For instance, mesopelagic species, widespread and numerous worldwide, are nutrient-dense sources and may contribute to new/novel marine ingredients for increased sustainability and feed security in the salmon producing industry [57].

## 5. Conclusions

One portion of farmed Atlantic salmon still provides equal amounts of EPA and DHA compared to wild salmon. However, the same portion would provide a high amount of fat/energy and omega-6 fatty acids but a lower amount of contaminants. Thus, farmed Atlantic salmon is a positive contribution to our diet regarding intake of marine omega-3 and reduced intake of contaminants but may be perceived as a negative contribution when energy restriction is taken into account.

## Figures and Tables

**Table 1:** Gutted weight (g), length (cm), fillet yield (%) and condition factor of wild (*n* = 23), farmed (*n* = 20), and escaped (*n* = 17) Atlantic salmon.

Parameter	Wild Salmon	Farmed Salmon	Escaped Salmon
Gutted weight (g)	4280 ± 1083 ^a^	4340 ± 264 ^a^	3588 ± 239 ^b^
Length (cm) ^2^	78.3 ± 7.3 ^a^	71.5 ± 2.2 ^ab^	70.4 ± 2.1 ^b^
Fillet yield (%) ^1^	61.4 ± 2.4	60.5 ± 1.7	60.9 ± 2.8
Condition factor	0.9 ± 0.1 ^c^	1.2 ± 0.1 ^a^	1.0 ± 0.1 ^b^

^1^ Fillet yield is based on the weight of one fillet multiplied by 2. ^2^ Length is measured from the nose of the fish to the tip of the caudal fin. Fulton’s condition factor is calculated as: (weight (g) × 100)/(length (cm)^3^). Values with different superscript letters in a row are significantly different (*p* < 0.05).

**Table 2:** Proximate water, protein and lipid composition (g/100 g of muscle) of wild (*n* = 23), farmed (*n* = 20), and escaped (*n* = 17) Atlantic salmon.

Parameter	Wild Salmon	Farmed Salmon	Escaped Salmon
Water	69.6 ± 2.4 ^a^	61.4 ± 1.6 ^c^	66.5 ± 1.7 ^b^
Ash	1.2 ± 0.1 ^a^	1.1 ± 0.1 ^b^	1.2 ± 0.0 ^ab^
Fat	6.0 ± 1.5 ^c^	17.9 ± 2.8 ^a^	12.0 ± 2.4 ^b^
Protein	16.2 ± 1.4 ^a^	15.4 ± 1.0 ^b^	15.8 ± 0.6 ^ab^

Values with different superscript letters in a row are significantly different (*p* < 0.05).

**Table 3:** Fatty acid composition (% of total FAs) and amount (g per 100 g of muscle) in wild (*n* = 23), farmed (*n* = 20) and escaped (*n* = 17) Atlantic salmon.

Fatty Acid	Wild Salmon		Farmed Salmon		Escaped Salmon	
	Composition (%)	Amount (g/100 g)	Composition (%)	Amount (g/100 g)	Composition (%)	Amount (g/100 g)
14:0	4.57 ± 0.81 ^a^	0.3 ± 0.1 ^a^	1.89 ± 0.06 ^b^	0.3 ± 0.1 ^b^	2.1 ± 0.4 ^c^	0.3 ± 0 ^a^
16:0	13.42 ± 0.92 ^a^	0.8 ± 0.2 ^a^	10.13 ± 0.37 ^b^	1.8 ± 0.3 ^b^	9.5 ± 1 ^c^	1.2 ± 0.2 ^c^
16:1n-7	4.77 ± 0.7 ^a^	0.3 ± 0.1 ^a^	2.2 ± 0.11 ^b^	0.4 ± 0.1 ^b^	2.5 ± 0.6 ^b^	0.3 ± 0 ^a^
18:0	2.85 ± 0.46	0.2 ± 0 ^a^	3.03 ± 0.13	0.5 ± 0.1 ^b^	2.6 ± 0.2 ^c^	0.3 ± 0.1 ^c^
18:1n-9	13.34 ± 1.64 ^a^	0.8 ± 0.2 ^a^	32.4 ± 0.56 ^b^	5.6 ± 1.1 ^b^	39.7 ± 4.8 ^c^	5 ± 1.2 ^b^
18:1n-7	3.02 ± 0.53 ^a^	0.2 ± 0.1 ^a^	2.53 ± 0.05 ^b^	0.5 ± 0.2 ^b^	3 ± 0.1 ^a^	0.4 ± 0.1 ^c^
18:2n-6 (LA)	1.35 ± 0.24 ^a^	0.1 ± 0 ^a^	14.38 ± 0.29 ^b^	2.5 ± 0.4 ^b^	12.8 ± 2.1 ^c^	1.6 ± 0.4 ^c^
18:3n-3 (ALA)	1.0 ± 0.2 ^a^	0.1 ± 0 ^a^	10.34 ± 0.37 ^b^	1.8 ± 0.3 ^b^	5.1 ± 0.9 ^c^	0.6 ± 0.2 ^c^
18:4n-3 (SDA)	3.13 ± 0.45 ^a^	0.2 ± 0.1 ^a^	0.81 ± 0.34 ^b^	0.1 ± 0.0 ^b^	1 ± 0.2 ^b^	0.1 ± 0 ^c^
20:1n-9	11.17 ± 1.14 ^a^	0.6 ± 0.2 ^a^	4.76 ± 1.69 ^b^	1.0 ± 0.1 ^b^	4.3 ± 1.1 ^b^	0.5 ± 0.1 ^c^
20:2n-6	n.d	n.d	1.69 ± 0.13 ^a^	0.3 ± 0.0 ^a^	1.3 ± 0.2 ^b^	0.2 ± 0 ^b^
20:4n-3	n.d	n.d	1.21 ± 0.12 ^a^	0.2 ± 0.0 ^a^	0.5 ± 0.1 ^b^	0.0 ± 0.0 ^b^
22:1n-11	11.99 ± 1.8 ^a^	0.7 ± 0.2 ^a^	1.23 ± 0.09 ^b^	0.2 ± 0.0 ^b^	2.5 ± 1.1 ^c^	0.3 ± 0 ^c^
22:1n-9	2.74 ± 0.24 ^a^	0.2 ± 0 ^a^	1.71 ± 0.07 ^b^	0.3 ± 0.1 ^b^	1.5 ± 0.2 ^b^	0.2 ± 0 ^c^
20:5n-3 (EPA)	6.58 ± 0.76 ^a^	0.4 ± 0.1 ^a^	2.64 ± 0.1 ^b^	0.5 ± 0.1 ^b^	2.6 ± 0.9	0.3 ± 0 ^c^
24:1n-9	0.95 ± 0.07	0.1 ± 0	n.d	n.d	n.d	n.d
22:5n-3 (DPA)	3 ± 0.36 ^a^	0.2 ± 0 ^a^	1.37 ± 0.14 ^b^	0.2 ± 0.0 ^b^	1.4 ± 0.3 ^b^	0.2 ± 0 ^a^
22:6n-3 (DHA)	14.56 ± 1.34 ^a^	0.8 ± 0.2 ^a^	4.94 ± 0.23 ^b^	0.9 ± 0.1 ^a^	5.6 ± 2.1 ^b^	0.7 ± 0.1 ^c^
SFA	20.84 ± 1.14 ^a^	1.2 ± 0.4 ^a^	15.05 ± 0.51 ^b^	2.7 ± 0.4 ^b^	14.2 ± 1.5 ^c^	1.7 ± 0.3 ^c^
MUFA	46.56 ± 3.81 ^a^	2.6 ± 0.8 ^a^	40.84 ± 0.7 ^b^	7.2 ± 1.2 ^b^	50.6 ± 1.0 ^c^	6.3 ± 1.3 ^c^
PUFA	30.95 ± 3.35 ^a^	1.7 ± 0.5 ^a^	41.93 ± 1.07 ^b^	7.4 ± 1.1 ^b^	32.8 ± 0.9 ^c^	4 ± 0.8 ^c^
LC-PUFA n-3	24.13 ± 1.55 ^a^	1.4 ± 0.4 ^a^	8.94 ± 0.5 ^b^	4.3 ± 0.6 ^b^	10.0 ± 3.2 ^c^	1.2 ± 0.1 ^c^
n-6/n-3	0.05 ± 0.01 ^a^	-	0.7 ± 0.01 ^b^	-	0.8 ± 0.2 ^b^	-

Values with different superscript letters in a row are significantly different (*p* ≤ 0.05). The fatty acids are represented by a notation indicating the number of carbon atoms and double bonds. ALA, alpha-linolenic acid; DHA, docosahexaenoic acid; DPA, docosapentaenoic acid; EPA, eicosapentaenoic acid; FA, fatty acid; LA, linoleic acid; MUFA, monounsaturated fatty acid; PUFA, polyunsaturated fatty acid; SFA, saturated fatty acid.

**Table 4:** Amino acid composition (mg/g filet) of wild (*n* = 23), farmed (*n* = 20), and escaped (*n* = 17) Atlantic salmon.

Amino Acid	Wild Salmon	Farmed Salmon	Escaped Salmon
Histidine	6.2 ± 0.7 ^a^	5.1 ± 0.3 ^b^	5.1 ± 0.3 ^b^
Threonine	9.6 ± 0.8 ^a^	9.2 ± 0.6 ^b^	9.4 ± 0.4 ^ab^
Isoleucine	8.8 ± 1.1 ^b^	9.5 ± 0.6 ^a^	9.7 ± 0.5 ^a^
Leucine	16.2 ± 1.3 ^a^	15.1 ± 1.0 ^b^	15.7 ± 0.6 ^ab^
Valine	10.2 ± 1.3 ^b^	11.0 ± 0.7 ^a^	11.1 ± 0.6 ^a^
Lysine	18.7 ± 1.6 ^a^	17.8 ± 1.2 ^b^	18.4 ± 0.7 ^ab^
Methionine	6.5 ± 0.5 ^a^	5.9 ±0.4 ^b^	6.1 ± 0.4 ^b^
Phenylalanine	8.6 ± 0.7 ^a^	8.1 ± 0.5 ^b^	8.3 ± 0.3 ^ab^
∑ Essential amino acids	84.9 ± 7.6 ^a^	81.7 ± 5.5 ^a^	83.9 ± 3.2 ^a^
Arginine	11.5 ± 1.7 ^ab^	11.4 ± 0.7 ^b^	11.8 ± 0.5 ^a^
Alanine	13.5 ±1.2 ^a^	11.7 ± 0.8 ^b^	12.31 ± 0.7 ^b^
Aspartic acid	15.3 ± 1.3 ^a^	14.8 ± 1.0 ^a^	15.3 ± 0.6 ^a^
Hydroxyproline	0.2 ± 0.2 ^b^	0.4 ± 0.0 ^a^	0.4 ± 0.2 ^a^
Beta alanine	2.1 ± 0.2 ^a^	2.1 ± 0.2 ^a^	1.9 ± 0.2 ^b^
Cysteine	0.6 ± 0.2 ^a^	0.6 ± 0.2 ^a^	0.6 ± 0.2 ^a^
Glycine	10.6 ± 1.1 ^a^	9.5 ± 0.6 ^b^	9.9 ± 0.6 ^b^
Glutamic acid	29.3 ± 2.6 ^a^	27.5 ± 1.9 ^b^	28.7 ± 1.1 ^ab^
Proline	7.5 ± 0.7 ^a^	7.3 ± 0.6 ^a^	7.2 ± 0.4 ^a^
Serine	8.1 ± 0.7 ^a^	7.5 ± 0.5 ^b^	7.7 ± 0.3 ^ab^
Tyrosine	7.5 ± 1.1 ^a^	6.8 ± 0.5 ^b^	7.0 ± 0.5 ^ab^
Taurine	0.6 ± 0.1 ^a^	0.5 ± 0.1 ^b^	0.6 ± 0.7 ^a^

Values with different superscript letters in a row are significantly different (*p* < 0.05).

**Table 5:** Contaminants in farmed salmon (*n* = 10), wild salmon (*n* = 12), and escaped salmon (*n* = 10) All values below or equal to level of quantification (LOQ) were set equal to LOQ.

Compound	Wild Salmon	Farmed Salmon	Escaped Salmon
Sum dl-PCB (pg TEQ/g)	0.95 ± 0.48 ^a^	0.17 ± 0.07 ^c^	0.44 ± 0.3 ^b^
Dioxins-furans (pg TEQ/g)	0.53 ± 0.12 ^a^	0.34 ± 0.04 ^b^	0.41 ± 0.11 ^b^
Dioxins + dl-PCB (pg TEQ/g)	1.48 ± 0.57 ^a^	0.51 ± 0.08 ^b^	0.85 ± 0.37 ^a^
Sum ICES-6 PCB (ng/g)	5.09 ± 0.83 ^a^	3.34 ± 0.46 ^c^	6.08 ± 0.45 ^b^
Mercury (µg/kg)	56.3 ± 12.9 ^a^	18.1 ± 1.5 ^c^	34.9 ± 3.1 ^b^
Lead (mg/kg)	0.01 ± 0.0	0.01 ± 0.0	0.01 ± 0.0
Cadmium (mg/kg)	0.01 ± 0.0	0.01 ± 0.0	0.01 ± 0.0
Arsenic (mg/kg)	2.56 ± 0.87 ^a^	0.86 ± 0.1 ^c^	1.68 ± 019 ^b^

Sum of PCDD/PCDF includes 2378-TCDD, 12378-PeCDD, 123478-HxCDD, 123678-HxCDD, 123789-HxCDD, 1234678-HpCDD and OCDD; 2378-TCDF, 12378-PeCDF, 23478-PeCDF, 123478-HxCDF, 123678-HxCDF, 123789-HxCDF, 234678-HxCDF, 1234678-HpCDF, 1234789-HpCD, OCDF. Sum ICES-6 PCB includes PCB28, 52, 101, 138, 153, and 180. Sum dl-PCB includes PCB 77, 81, 126, 169, 105, 114, 118, 123, 156, 157, 167, and 189. Values with different superscript letters in a row are significantly different (*p* < 0.05).

## References

[1] Hoegh-Guldberg O., Caldeira K., Chopin T., Gaines S., Haugan P., Hemer M., Howard J., Konar M., Krause-Jensen D., Lindstad E. (2019). The Ocean as a Solution to Climate Change: Five Opportunities for Action. https://www.wri.org/events/2019/10/ocean-solution-climate-change-5-opportunities-action.

[2] Hilborn R., Banobi J., Hall S.J., Pucylowski T., Walsworth T.E. (2018). The environmental cost of animal source foods. Front. Ecol. Environ..

[3] Chandra A., Røsjø H., Eide I.A., Vigen T., Ihle-Hansen H., Orstad E.B., Rønning O.M., Lyngbakken M.N., Berge T., Schmidt E.B. (2020). Plasma marine n-3 polyunsaturated fatty acids and cardiovascular risk factors: Data from the ACE 1950 study. Eur. J. Nutr..

[4] Rimm E.B., Appel L.J., Chiuve S.E., Djousse L., Engler M.B., Kris-Etherton P.M., Mozaffarian D., Siscovick D.S., Lichtenstein A.H. (2018). Seafood long-chain n-3 polyunsaturated fatty acids and cardiovascular disease: A science advisory from the American Heart Association. Circulation.

[5] Afshin A., Sur P.J., Fay K.A., Cornaby L., Ferrara G., Salama J.S., Mullany E.C., Abate K.H., Abbafati C., Abebe Z. (2019). Health effects of dietary risks in 195 countries, 1990-2017: A systematic analysis for the Global Burden of Disease Study 2017. Lancet.

[6] Maehre H.K., Jensen I.J., Elvevoll E.O., Eilertsen K.E. (2015). Omega-3 fatty acids and cardiovascular diseases: Effects, Mechanisms and dietary relevance. Int. J. Mol. Sci..

[7] Innes J.K., Calder P.C. (2020). Marine omega-3 (n-3) fatty acids for cardiovascular health: An update for 2020. Int. J. Mol. Sci..

[8] Aung T., Halsey J., Kromhout D., Gerstein H.C., Marchioli R., Tavazzi L., Geleijnse J.M., Rauch B., Ness A., Galan P. (2018). Associations of omega-3 fatty acid supplement use with cardiovascular disease risks meta-analysis of 10 trials involving 77 917 individuals. JAMA Cardiol..

[9] Jensen I.J., Walquist M., Liaset B., Elvevoll E.O., Eilertsen K.E. (2016). Dietary intake of cod and scallop reduces atherosclerotic burden in female apolipoprotein E-deficient mice fed a Western-type high fat diet for 13 weeks. Nutr. Metab..

[10] Food and Agriculture Organization of the United Nations (FAO), World Health Organization (WHO) (2011). Report of the Joint FAO/WHO Expert Consultation on the Risks and Benefits of Fish Consumption.

[11] Malisch R., Kotz A. (2014). Dioxins and PCBs in feed and food—Review from European perspective. Sci. Total Environ..

[12] Larsen J.C. (2006). Risk assessments of polychlorinated dibenzo-p-dioxins, polychlorinated dibenzofurans, and dioxin-like polychlorinated biphenyls in food. Mol. Nutr. Food Res..

[13] Nogara P.A., Farina M., Aschner M., Rocha J.B. (2019). Mercury in our food. Chem. Res..

[14] United Nations Treaty Collection. 15. Stockholm Convention on Persistent Organic Pollutants. https://treaties.un.org/Pages/ViewDetails.aspx?src=IND&mtdsg_no=XXVII-15&chapter=27&clang=_en#1.

[15] World Health Organization (WHO), Food and Agriculture Organization of the United Nations (FAO) (2018). Code of practice for the prevention and reduction of dioxins, dioxin-like PCBs and non-dioxin-like PCBs in food and feed. Codex Alimentarius Commission.

[16] Sissener N.H. (2018). Are we what we eat? Changes to the feed fatty acid composition of farmed salmon and its effects through the food chain. J. Exp. Biol..

[17] Nostbakken O.J., Hove H.T., Duinker A., Lundebye A.K., Berntssen M.H.G., Hannisdal R., Lunestad B.T., Maage A., Madsen L., Torstensen B.E. (2015). Contaminant levels in Norwegian farmed Atlantic salmon (*Salmo salar*) in the 13-year period from 1999 to 2011. Environ. Int..

[18] Scientific Committee for Food and the Environment (VKM) (2014). Benefit-Risk Assessment of Fish and Fish Products in the Norwegian Diet—An Update.

[19] EFSA (2018). Risk for animal and human health related to the presence of dioxins and dioxin-like PCBs in feed and food. EFSA J..

[20] Van den Berg M., Birnbaum L.S., Denison M., De Vito M., Farland W., Feeley M., Fiedler H., Hakansson H., Hanberg A., Haws L. (2006). The 2005 World Health Organization reevaluation of human and mammalian toxic equivalency factors for dioxins and dioxin-like compounds. Toxicol. Sci..

[21] Iversen A., Asche F., Hermansen Ø., Nystøyl R. (2020). Production cost and competitiveness in major salmon farming countries 2003–2018. Aquaculture.

[22] Jensen I.J., Maehre H.K., Tømmerås S., Eilertsen K.E., Olsen R.L., Elvevoll E.O. (2012). Farmed Atlantic salmon (*Salmo salar* L.) is a good source of long chain omega-3 fatty acids. Nut. Bull..

[23] Lund R.A., Hansen L.P. (1991). Identification of wild and reared Atlantic salmon, *Salmo salar* L., using scale characters. Aquac. Fish. Manage.

[24] Fiske P., Lund R.A., Hansen L.P., Cadrin S.X., Friedland K.D., Waldman J.R.E. (2005). Identifying fish farm escapees. Stock Identification Methods: Applications in Fishery Science.

[25] Folch J., Lees M., Sloane Stanley G.H. (1957). A simple method for the isolation and purification of total lipides from animal tissue. J. Biol. Chem..

[26] Maehre H.K., Dalheim L., Edvinsen G.K., Elvevoll E.O., Jensen I.J. (2018). Protein Determination—Method Matters. Foods.

[27] AOAC International (2019). Official Methods of Analysis of AOAC International.

[28] Maehre H.K., Hamre K., Elvevoll E.O. (2013). Nutrient evaluation of rotifers and zooplankton: Feed for marine fish larvae. Aquac. Nutr..

[29] Stoffel W., Chu F., Ahrens E.H. (1959). Analysis of long-chain fatty acids by gas-liquid chromatography—Micromethod for preparation of methyl esters. Anal. Chem..

[30] Moore S., Stein W.H. (1963). Chromatographic determination of amino acids by the use of automatic recording equipment. Meth. Enzymol..

[31] U.S. Environmental Protection Agency (1994). Method 1613: Tetra-Through Octa-Chlorinated Dioxins and Furans by Isotope Dilution HRGC/HRMS.

[32] U.S. Environmental Protection Agency (1999). Method 1668C Chlorinated Biphenyl Congeners in Water, Soil, Sediment, Biosolids, and Tissue by HRGC/HRMS.

[33] U.S. Environmental Protection Agency (1998). Method 7473 (SW-846): Mercury in Solids and Solutions by Thermal Decomposition, Amalgamation, and Atomic Absorption Spectrophotometry.

[34] World Health Organization (WHO) (2007). Protein and Amino Acid Requirements in Human Nutrition. Report of a Joint WHO/FAO/UNU Expert Consultation.

[35] Forseth T., Barlaup B.T., Finstad B., Fiske P., Gjøsæter H., Falkegård M., Hindar A., Mo T.A., Rikardsen A.H., Thorstad E.B. (2017). The major threats to Atlantic salmon in Norway. ICES J. Mar. Sci..

[36] Glover K.A., Urdal K., Næsje T., Skoglund H., Florø-Larsen B., Otterå H., Fiske P., Heino M., Aronsen T., Sægrov H. (2019). Domesticated escapees on the run: The second-generation monitoring programme reports the numbers and proportions of farmed Atlantic salmon in> 200 Norwegian rivers annually. ICES J. Mar. Sci..

[37] Lundebye A.K., Lock E.J., Rasinger J.D., Nøstbakken O.J., Hannisdal R., Karlsbakk E., Wennevik V., Madhun A.S., Madsen L., Graff I.E. (2017). Lower levels of persistent organic pollutants, metals and the marine omega 3-fatty acid DHA in farmed compared to wild atlantic salmon (*Salmo salar*). Environ. Res..

[38] Skilbrei O.T., Normann E., Meier S., Olsen R.E. (2015). Use of fatty acid profiles to monitor the escape history of farmed Atlantic salmon. Aquac. Environ. Interact..

[39] Bicskei B., Bron J.E., Glover K.A., Taggart J.B. (2014). A comparison of gene transcription profiles of domesticated and wild Atlantic salmon (*Salmo salar* L.) at early life stages, reared under controlled conditions. BMC Genom..

[40] Aas T.S., Ytrestøyl T., Åsgård T. (2019). Utilization of feed resources in the production of Atlantic salmon (*Salmo salar*) in Norway: An update for 2016. Aquac. Rep..

[41] Bell J.G., McEvoy J., Webster J.L., McGhee F., Millar R.M., Sargent J.R. (1998). Flesh Lipid and Carotenoid Composition of Scottish Farmed Atlantic Salmon (*Salmo Salar*). J Agric. Food Chem..

[42] Bendiksen E.Å., Johnsen C.A., Olsen H.J., Jobling M. (2011). Sustainable aquafeeds: Progress towards reduced reliance upon marine ingredients in diets for farmed Atlantic salmon (*Salmo salar* L.). Aquaculture.

[43] Ytrestøyl T., Aas T.S., Åsgård T. (2015). Utilisation of feed resources in production of Atlantic salmon (*Salmo salar*) in Norway. Aquaculture.

[44] Colombo S.M., Mazal X. (2020). Investigation of the nutritional composition of different types of salmon available to Canadian consumers. J. Agric. Food Chem..

[45] Tørris C., Molin M., Småstuen M.C. (2017). Lean fish consumption is associated with beneficial changes in the metabolic syndrome components: A 13-year follow-up study from the Norwegian Tromsø Study. Nutrients.

[46] Sissener N.H., Julshamn K., Espe M., Lunestad B.T., Hemre G.-I., Waagbø R., Måge A. (2013). Surveillance of selected nutrients, additives and undesirables in commercial Norwegian fish feeds in the years 2000–2010. Aquac. Nutr..

[47] Friedman N. (1996). Nutritional value of protein from different food sources. J. Agric. Food Chem..

[48] EFSA EEFSA sets European Dietary Reference Values for Nutrient Intakes. https://efsa.onlinelibrary.wiley.com/doi/epdf/10.2903/j.efsa.2010.1461.

[49] Simopoulos A.P. (2009). Evolutionary Aspects of the Dietary Omega-6: Omega-3 Fatty Acid Ratio: Medical Implications. World Rev. Nutr. Diet..

[50] (2012). European Food Safety Authority (EFSA) Update of the monitoring of levels of dioxins and PCBs in food and feed. EFSA J..

[51] Totland T.H., Melnæs B.K., Lundberg-Hallén N., Helland-Kigen K.M., Lund-Blix N.A., Myhre J.B., Johansen A.M.W., Løken E.B., Andersen L.F. (2012). Norkost 3. En Landsomfattende Kostholdsundersøkelse Blant Menn og Kvinner i Norge i Alderen 18-70 år. 2010–2011.

[52] Norway S. Elvefiske. https://www.ssb.no/jord-skog-jakt-og-fiskeri/statistikker/elvefiske.

[53] Van Ede K.I., Van Duursen M.B.M., van den Berg M. (2016). Evaluation of relative effect potencies (REPs) for dioxin-like compounds to derive systemic or human-specific TEFs to improve human risk. Arch. Toxicol..

[54] Horvat M., Worsfold P., Townshend A., Poole C. (2005). Mercury. Encyclopedia of Analytical Science.

[55] (2006). Commission regulation (EC) No 1881/2006 of 19 December 2006 Setting Maximum Levels for Certain Contaminants in Foodstuffs. https://eur-lex.europa.eu/legal-content/EN/ALL/?uri=CELEX%3A32006R1881.

[56] Food and Agriculture Organization of the United Nations (FAO) (2020). The State of World Fisheries and Aquaculture: Sustainability in Action.

[57] Alvheim A.R., Kjellevold M., Strand E., Sanden M., QWiech M. (2020). Mesopelagic species and their potential contribution to food and feed security—A case study from Norway. Foods.

